# Chitosan nanoparticles: A positive modulator of innate immune responses in plants

**DOI:** 10.1038/srep15195

**Published:** 2015-10-16

**Authors:** Swarnendu Chandra, Nilanjan Chakraborty, Adhiraj Dasgupta, Joy Sarkar, Koustubh Panda, Krishnendu Acharya

**Affiliations:** 1 Molecular and Applied Mycology and Plant Pathology Laboratory, Department of Botany, University of Calcutta, 35, Ballygunge Circular Road, Kolkata-700019 India; 2 Department of Biotechnology & Guha Center for Genetic Engineering & Biotechnology, University of Calcutta, 35, Ballygunge Circular Road, Kolkata-700019, India

## Abstract

The immunomodulatory role of the natural biopolymer, chitosan, has already been demonstrated in plants, whilst its nanoparticles have only been examined for biomedical applications. In our present study, we have investigated the possible ability and mechanism of chitosan nanoparticles (CNP) to induce and augment immune responses in plants. CNP-treatment of leaves produced significant improvement in the plant’s innate immune response through induction of defense enzyme activity, upregulation of defense related genes including that of several antioxidant enzymes as well as elevation of the levels of total phenolics. It is also possible that the extracellular localization of CNP may also play a role in the observed upregulation of defense response in plants. Nitric oxide (NO), an important signaling molecule in plant defense, was also observed to increase following CNP treatment. However, such CNP-mediated immuno-stimulation was significantly mitigated when NO production was inhibited, indicating a possible role of NO in such immune induction. Taken together, our results suggest that CNP may be used as a more effective phytosanitary or disease control agent compared to natural chitosan for sustainable organic cultivation.

The growing interest and demand for organic or non-polluted food/crop from the more health conscious consumers and the challenge posed by the evolving adaptability of phyto-pathogens due to uncontrolled use of synthetic chemicals, have led to the exploration of alternative crop protection strategies in recent times. The search for such alternative disease management strategies supported by the advancement of nanotechnology, have also paved for application studies of nanomaterials as a potential candidate for disease control in plants. Application of different metal nanoparticles (NP) have already generated a quantifiable data against different phyto-pathogens, but the unstable and toxic nature of metal NP have raised serious concerns with respect to their use^1^,^2^.

Chitosan has been widely used for cosmetic and material based applications. However, in recent times biomedicine and agriculture have also witnessed a growing interest in chitosan as a therapeutic agent. In the plant system, chitosan has been reported to induce multifaceted disease resistance^3^. This natural biopolymer is known for its unique properties like, biodegradability, nontoxicity and antimicrobial activity, thus popularizing its use as an elicitor molecule for different host-pathogen interaction studies^2^. Such unique properties of the chitosan biopolymer can be further enhanced by using it in the form of nanoparticles (CNP), as in this form it can instill different biological activities with altered physicochemical properties like size, surface area, cationic nature etc. Its unique biocompatibility, biodegradability and low toxicity apparently makes CNP a more effective nano-delivery system compared to its close counterparts^4^. In fact, the CNP are not only more stable and less toxic, but also requires the use of simple preparative methods which make them a versatile and user-friendly drug delivery agent^5^. Apart from their biomedical applications, CNP have only been reported to have antifungal properties against different phyto-pathogens^1^. In fact, nanoparticles by themselves can negotiate cell walls and membranes far more effectively compared to the core molecules they are prepared from. This partly explains why CNP were observed to demonstrate better immune stimulation compared to chitosan itself in our study.

Despite having been used in the field of agriculture, chitosan has never been applied in the form of a nanoparticle for inducing innate immunity in plants. Our present study demonstrates the unique ability of chitosan nanoparticles (CNP) to boost innate immunity in plants and also indicates the possible involvement of nitric oxide (NO) in eliciting such response. Our results convincingly establish CNP as a potential biocompatible plant defense tool for better control of plant diseases in the future.

## Results

### Characterization of CNP

**Figure 1:**
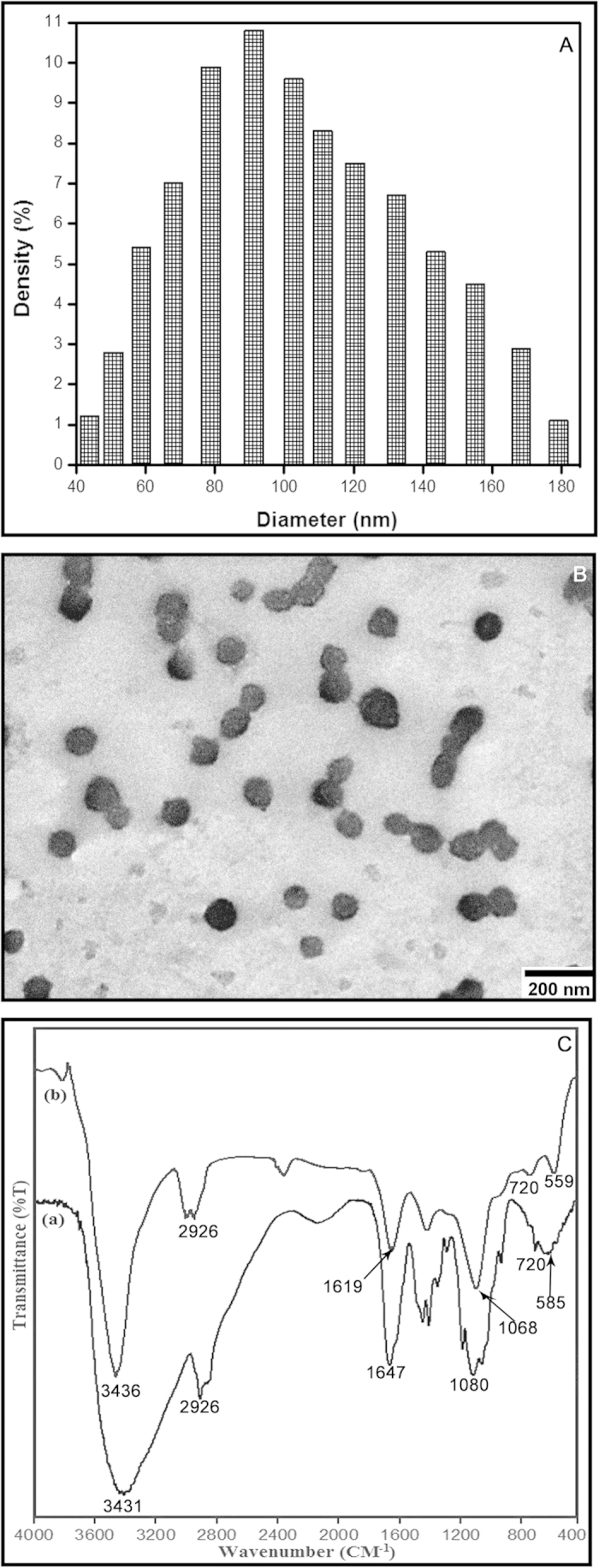
Characterization of CNP. (**A**) Histogram of particle size distribution as obtained from light scattering of the chitosan nanoparticles, (**B**) Transmission electron micrograph (TEM) of chitosan nanoparticles (**C**) FTIR absorption spectra of (a) pure chitosan and (b) CNP. Results are representative of three independent experiments done under similar conditions.

The size distribution profile of the synthesized nanoparticles was found to be in the range of 40–180 nm (Fig. 1A). Further characterization by Transmission Electron Microscopy (TEM) showed that the single or the aggregated nanoparticles on the carbon coated copper grids were more or less spherical in shape with an average diameter of 90 ± 5 nm (Fig. 1B). In fact, particle size obtained from such TEM analysis is seemingly more accurate than the size measured by the dynamic light scattering method (DLS). This is because, in TEM analysis particles are measured in their dry state, whereas in DLS, the nanoparticles being in their hydrated state project an apparently larger hydrodynamic diameter due to the presence of solvent layers^6^.

FTIR absorption spectra of vacuum-dried pure chitosan and chitosan nanoparticles are shown in the Fig. 1(C). The C-H stretching vibrations are manifested through strong peaks at around 2926 cm^−1 ^7. Acetyl groups characteristically absorbed in the range 1300–1100 cm^−1^, as seen in both the chitosan and CNP spectrum respectively. The symmetric stretch of C-O-C was observed around 1080–1060 cm^−1 ^7. The peaks visible in between 500 and 749 cm^−1^ signifies the presence of R−CH group^8^. As seen in both the spectra, the strong peaks in the range 3450–3200 cm^−1^ correspond to combined peaks of hydroxyl and intra-molecular hydrogen bonding. The broadness of the peak at this region might be attributed to contributions from N-H bond stretches^7^. In CNP, a shift from 3436 to 3431 cm^−1^ was observed, and the peak at 3431 cm^−1^ of pure chitosan compound was found wider, which indicated extensive hydrogen bonding^9^. The characteristic CONH_2_ peak of chitosan observed around 1647 cm^−1^, was found to shift to 1619 cm^−1^ in the CNP, which could be due to the tripolyphosphate cross-linking with the ammonium groups in CNP^10^. This cross-linking between the polyphosphates and the ammonium groups might be important for the inter- and intra-molecular interactions that are reflected in our results^10^.

### Effect of chitosan and CNP on defense enzymes

The efficacy of chitosan in induction of plant defense is perhaps a well-documented fact^3^, whereas CNP have mainly been studied for biomedical applications. Only recently, an *in vitro* study reported antimicrobial activity of CNP against phytopathogens^1^. In our present study, we demonstrate for the first time the capability of CNP to produce significantly higher defense response in plants compared to that accomplished through use of natural chitosan, even when such CNP are administered at ten times more diluted dosage compared to that of chitosan (standardized unpublished data). Leaves of *Camellia sinensis* were separately treated with chitosan and CNP, and the defense enzyme activities were estimated after an incubation period of 24 h at room temperature. The increased activity of the defense enzymes were in the order of peroxidase (PO) > polyphenol oxidase (PPO) > phenylalanine ammonia lyase (PAL) > β-1,3-glucanase. Treatment with chitosan showed 29.26% increase in β-1,3-glucanase and more than 0.8 fold increase in PAL activity over the untreated control. Simultaneously, more than 3 fold increase in PO and 2 fold increase in PPO activity in the chitosan treated leaves were also observed. In contrast, CNP treated leaves showed significant (*p* < *0.05*) increase in accumulation of PO, PPO and PAL (more than 4, 3.5 and 2 fold increase respectively), whereas the β-1,3-glucanase levels increased by about 34% (Table 1).

### Effect of chitosan and CNP on antioxidant enzymes

To examine the role of chitosan and CNP treatment on ROS inhibition in the *C. sinensis* leaves, we measured the activity of two important antioxidant enzymes superoxide dismutase (SOD) and catalase (CAT). Increase in the levels of SOD and CAT activity in the chitosan and CNP-treated sets were observed after 24 h against the water-treated control set. About 33% and 40% increase in SOD and CAT activities were observed in chitosan treated sets, whereas the same enzymes showed around 41% and 49% induction in enzyme activity respectively following CNP treatment (Table 1).

### Effect of chitosan and CNP on levels of phenols and flavonoids

Phenols are considered to be one of the most important natural defense mediators in plants. Thus, in the course of evaluation of induction in plant defense responses, we studied the total phenolic levels in both chitosan and CNP treated leaves. Total phenolic accumulation after 24 h exposure to chitosan and CNP, respectively induced around 20% and 24% increase over the control sets (Table 1). The induction of total phenol content in the CNP treated leaves was found to be 3.5% higher than that of chitosan treated ones.

Among all phenols, flavonoids are most abundant in tea leaves, while among such flavonoids, flavan-3-ols constitute the major component^11^. As the involvement of these flavonoids in plant defense is well documented^12^,^13^, we investigated the possible accumulation of flavonoids in the treated leaves by High Performance Liquid Chromatography (HPLC). The application of chitosan and CNP showed significant (*p* < *0.05*) increase in accumulation of gallic acid (GA), epicatechin (EC), epigallocatechin (EGC), epigallocatechin gallate (EGCG) and caffeine in the cell lysates of the treated leaves compared to that of the untreated controls (Table 2).

### Effect of chitosan and CNP on defense-related gene expression

**Figure 2:**
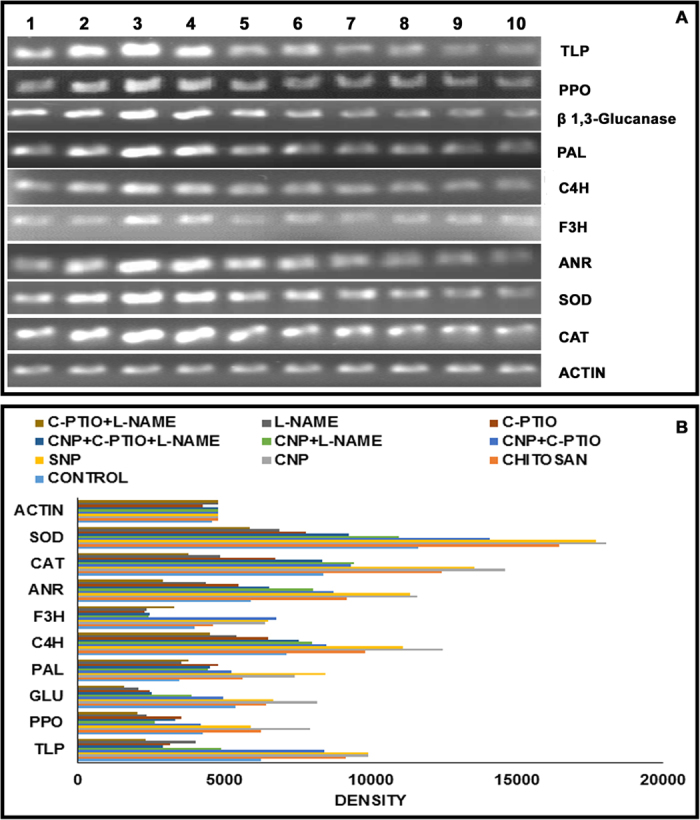
Semi-quantitative RT-PCR analyses. (**A**) Expression of different defense related genes (TLP, PPO, β-1,3-glucanase, PAL), antioxidant enzyme-coding genes (SOD, CAT) and genes of flavonoid biosynthesis pathway (C4H, F3H, ANR) are represented in the following order of treatment: Lane (1) control (water); (2) chitosan (0.01%); (3) CNP (0.001%); (4) SNP (100 mM); (5) CNP (0.001%)+C-PTIO (100 μM); (6) CNP (0.001%)+L-NAME (10 μM); (7) CNP (0.001%)+L-NAME (10 μM)+C-PTIO (100 μM); (8) C-PTIO (100 μM); (9) L-NAME (10 μM); (10) L-NAME (10 μM)+C-PTIO (100 μM). Actin band represents internal controls for sample loading. (**B**) Densitometric analysis of the gene expressions.

Changes in expression of different defense related genes including genes of antioxidant enzymes and some important intermediary flavonoid biosynthesis genes in the chitosan and CNP-treated sets were analysed by semi-quantitative RT-PCR. Induced defense enzyme activities were also reflected in the transcript analysis study. As shown in Fig. 2, the differential alteration of PPO, β-1,3-glucanase, PAL and thaumatin like protein (TLP) gene expressions in both the chitosan and CNP treated sets were markedly higher, than in the control. In comparison to chitosan treatment, leaves of the CNP treated sets showed higher expression of the same defense related genes and enzymes. To predict the role of chitosan and CNP in ROS inhibition we studied the gene expression of two important antioxidant enzymes SOD and CAT. The mRNA expressions of the two genes in the 24 h treated leaves were considerably higher than that of the untreated controls (Fig. 2). Such results indicate that SOD and CAT potentially provide protection to the plants against different oxidative stresses. Furthermore, the expression analysis of important intermediary genes in flavonoid biosynthesis pathway revealed an overexpression of cinnamate 4-hydroxylase (C4H), flavonoid 3-hydroxylase (F3H) and anthocyanidin reductase (ANR) genes (Fig. 2) in chitosan and CNP treated leaves. These findings support the higher accumulation of different flavonoids in the cell lysates of the chitosan and CNP treated leaves and also indicate a secondary defense induction in the treated plants.

### Effect of chitosan and CNP in cellular NO generat**ion**

**Figure 3:**
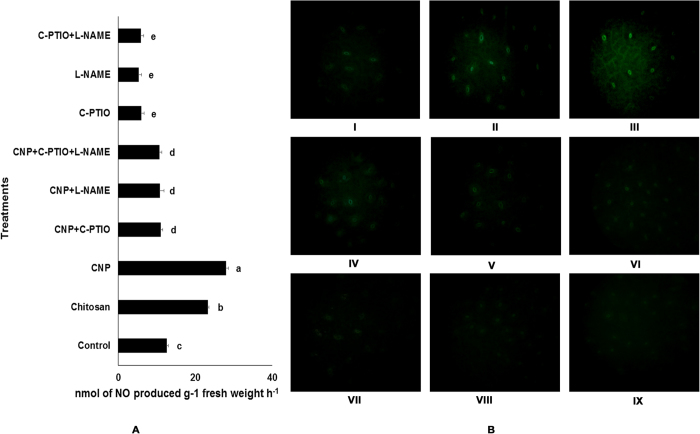
Effect of exogenous treatment of Chitosan (0.1%) and CNP (0.01%) along with different NO modulators on the extent of NO production in leaves of *C. sinensis*. (**A**) Spectrophotometric analysis of NO production; (**B**) Real-time determination of NO in leaf epidermal cells by DAF-2DA staining. NO generation was measured from the green fluorescence intensity of the stained leaves. Panels represent following order of treatment: (I) control (water); (II) chitosan (0.01%); (III) CNP (0.001%); (IV) SNP (100 μM); (V) CNP (0.001%)+C-PTIO (100 μM); (VI) CNP (0.001%)+L-NAME (10 μM); (VII) CNP (0.001%)+L-NAME (10 μM)+C-PTIO (100 μM); (VII) C-PTIO (100 μM); (VIII) L-NAME (10 μM); (IX) L-NAME (10 μM)+C-PTIO (100 μM). Results are mean ± SD of three separate experiments done in triplicate. Different letters in the bar graph indicate significant difference (*p* < *0.05*) from the control set using Duncan’s multiple range test whereas same letter denotes no significant difference between the groups.

It has already been established that NO directly or indirectly modulates the immune responses in plants and also plays a key role in stimulation of defense response in the chitosan-treated plants^14^,^15^. To investigate whether defense augmentation in our model plant by chitosan and CNP is NO mediated, we evaluated the levels of NO release in both chitosan and CNP-treated leaves and compared them with the untreated control. In both the treatments, an elevated level of NO production was observed when compared to the control. Results show significant increase in NO generation in the CNP treated leaves compared to both control and chitosan treated leaves (Fig. 3A). Around 1.8 and 2.2 fold increase in NO accumulation was observed in the chitosan and CNP-treated sets respectively. To further determine the real-time NO generation in chitosan and CNP-treated leaves we stained the treated leaves with DAF-2DA. As the intensity of the green fluorescence detected in the treated leaves through microscopic examination is indicative of the amount of NO generated in the cells real-time, we observed that both the chitosan and CNP-treated leaves showed much higher levels of NO generation compared to the untreated leaves (Fig. 3B).

### Effect of NO donor and NO modulators on defense response

Effective induction of defense response and production of NO in the CNP treated cells are evident from the above results. To further understand the involvement of NO in the observed CNP-mediated defense induction, we created NO surplus and NO depleted environments in the target leaves. In situations where NO was readily available to the plant cells [i.e., using sodium nitroprusside (SNP)], a significant elevated levels of defense enzymes namely, PO (4 fold), PPO (3.7 fold), β-1,3-glucanase (1.3 fold) and PAL (2.2 fold) along with the induced activity of antioxidant enzymes SOD (1.7 fold) and CAT (1.85 fold) was observed (Table 1). Also, a concurrent higher accumulation of total phenolics (1.3 fold) and different flavonoids were detected (Tables 2 & 3). Simultaneously, higher expression of defense related genes and antioxidant enzyme-coding genes as well as the genes of flavonoid biosynthesis pathway were also witnessed (Fig. 2).

Although the generation of NO was significantly higher in CNP-treated cells, co-treatment of CNP with the potent NO inhibitors, NG-nitro-L-arginine methyl ester (L-NAME) or 2-(4-Carboxyphenyl)-4, 4, 5, 5-tetramethylimidazoline-1-oxyl-3-oxide (C-PTIO) in different combinations, limited the NO generation considerably (Fig. 3). In fact, almost 12–15% decrease in NO generation in the CNP+C-PTIO, CNP+L-NAME and CNP+C-PTIO+L-NAME sets were detected (Fig. 3A). When lower epidermal peels from the afore-mentioned sets were visualised under the microscope, no or very little NO-related fluorescence were visualized (Fig. 3B). However, accumulation of defense enzymes, antioxidant enzymes, total phenolic contents (Table 1) and different flavonoids (Table 2) along with the expression profiles of different defense related genes, including genes of antioxidant enzymes and some important intermediary flavonoid biosynthesis pathway genes was found to be more or less similar to the observed basal or control level (Fig. 2). Similarly, generation of NO and the elevation of defense responses were also found to be limited in the NO deprived environments (i.e. using L-NAME and C-PTIO alone or in combinations). In the C-PTIO and L-NAME treated sets, significant decrease in NO generation were observed (Fig. 3A). Microscopic observations revealed almost no fluorescence signals from the lower epidermal peels of the above mentioned sets indicating NO release (Fig. 3B). A significant decrease in the activity of defense enzymes and antioxidant enzymes along with the reduction in total phenolic accumulation were also observed in the C-PTIO and L-NAME treated sets (Table 1). Cell lysates of different sets treated with different inhibitors alone or in different combinations with CNP, when analysed by HPLC, showed a considerable decrease in the accumulation of different flavon-3-ol compounds (Table 2). Expression profiles of different defense related genes, genes of antioxidant enzymes and some important intermediary genes of flavonoid biosynthesis pathway were also found to be down regulated in the NO deprived sets (Fig. 2).

### Bioaccumulation of chitosan and CNP in leaves

**Figure 4:**
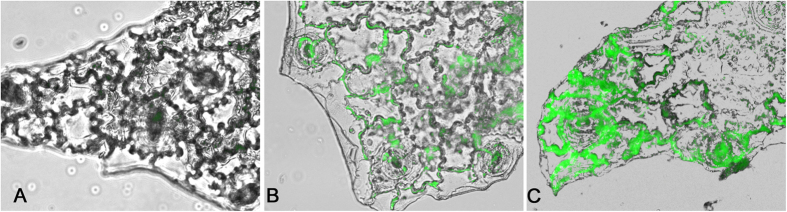
Fluorescence microscopic depiction of relative bioaccumulation and bioaccessibility of Chitosan (CHP) and Chitosan Nanoparticles (CNP) in treated leaves. The lower epidermal peals incubated with FITC tagged (**B**) Chitosan (CHT-FITC) and (**C**) CNP (CNP-FITC) manifest intense green fluorescence around the cell walls against water treated counterparts (**A**) Control. Figures are representative of three independent experiments done under similar conditions.

*In vitro* cellular uptake of chitosan and CNP in the cells of the treated leaves were studied using FITC-tagged chitosan (CHT-FITC) and CNP (CNP-FITC). The fluorescent microscopic images of such leaves are depicted in Fig. 4. Both CHT-FITC and CNP-FITC treated sets showed intensely illuminated cell walls of the subsidiary cells in the lower epidermal peels compared to the FITC treated control set. Binding of CHT-FITC was generally lower than that of CNP-FITC prepared from the same chitosan sample. Cells incubated with CNP-FITC at ambient temperature exhibited a thicker layer of stronger fluorescence around the cell walls than cells incubated under the same conditions with CHT-FITC. As control sets were incubated in water containing FITC only, no such fluorescence signal was observed in epidermal cells.

## Discussion

Natural defense response of plants against pathogenesis depends upon early recognition of pathogens. In fact, over the passage of evolution, plants have developed various strategies to combat different evolving pathogens. This induction of natural defense response includes over expression of different defense related genes and enzymes, increased accumulation of phenolic compounds, cell wall synthesis etc.^16^. Plants treated with different biotic elicitor molecules have been shown to induce this innate immune response by mimicking different pathogens^17^.

Chitosan, a natural polymer, has been reported as an effective biotic elicitor that induces the systemic resistance in plants^18^. However, the relative insolubility of chitosan in aqueous medium limits its use as an antifungal agent^2^. So far, chitosan in the form of nanoparticles has not been examined for its phyto-immunogenic activity as much as its natural form. In a previous *in vitro* study chitosan nanoparticles (CNP) showed significant antimicrobial activity against plant pathogens^1^. In our present work we demonstrate that CNP can also act as an effective plant defense elicitor in an *in vivo* setting, and can accomplish far better immunomodulatory efficacy compared to natural chitosan, that too at almost ten times lower dose compared to what is required for chitosan.

Although we began our study by applying both chitosan and CNP on the leaves of live plants during the preliminary stage of our study, we subsequently found that the results obtained with leaves collected from such treated whole plants after 24 h were almost similar to that obtained with freshly excised leaves which were treated with chitosan and CNP for the same period. Moreover, several experimental designs in the present study like monitoring dynamic release of NO by the CNP-treated leaves through fluorescence imaging along with modulating NO levels with the use of NO-scavenger and inhibitor could not have been accomplished using whole plants both for technical as well as factors of cost (as the chemicals involved in such treatment are quite costly and constraining to deploy in large amounts). Most importantly, since the primary defense response to chitosan or CNP between the *ex-vivo* (whole plants) and *in vivo* (freshly excised leaves) experiments were same we could afford to conduct the whole study *ex-vivo* using excised leaves of *Camellia sinensis* (tea), being confident of the overall physiological relevance of such work with respect to whole plants (*in vivo*).

The application of chitosan and CNP on tea leaves remarkably elevated the defense response in the tea plant. Analysis of defense enzymes showed higher accumulation of PO, PPO, β-1,3-glucanase, PAL in the CNP treated leaves when compared to the chitosan treated counterparts. Also, transcript analysis of CNP treated leaves revealed higher expression of PPO, β-1,3-glucanase, PAL and TLP genes than that treated with chitosan. Among these enzymes, PO and PPO are known to be involved in the lignin biosynthesis pathway^19^, which enhances protection against different pathogens by strengthening the plant’s cell wall barrier^20^. On the other hand the pathogenesis related (PR) protein, β-1,3-glucanase is well known for its antifungal activity. Induced expression of β-1,3-glucanase would not only contribute to provide increased protection against pathogens, but would also enhance diverse physiological roles including cell division, flower formation and seed maturation^21^. Thus induction of expression of β-1,3-glucanase by chitosan or CNP treatment might become effective at the time of pathogen invasion as this enzyme is directly involved in the hydrolyzing of fungal cell wall glucans. Another PR protein, TLP has also been reported for its antifungal and anti-insecticidal activity. Therefore, induced activity of TLP can expectedly help enhance immunity against pathogens including insects. Thus, several reports on chitosan-mediated induction of plant defense has been reported^3^,^22^. Application of chitosan have been shown to induce increased expression of PO and PPO^23^, PAL^24^, and β-1,3-glucanase^25^ in different host plants.

Our cellular localization studies also showed that both chitosan and CNP bind to the cell extracellularly. More intense localization of fluorescence caused by FITC tagged to CNP might be correlated with our results related to high level of defense response in CNP treated leaves when compared to that of chitosan. Extracellular localization of chitosan in suspension culture of tobacco leaf cells was also reported by Zhao *et al.*^26^ where fluorescent labelled oligochitosan was found to be present around the cell walls.

PAL is first enzyme in the phenylpropanoid biosynthesis pathway and it is involved in the production of phenolics and phytoalexins^27^. The role of phenolic substances in disease resistance^28^ and their accumulation by the phenyl propanoid pathway due to various elicitor treatments have already been documented earlier^16^,^29^. In this work, tea leaves treated with CNP showed higher accumulation of phenolic compounds than chitosan treated ones, which could be a direct result of the up-regulation of PAL activity in the CNP treated sets. This up-regulation was reaffirmed by the assessment of the induced expression of C4H, F3H and ANR genes in the chitosan and CNP treated leaves. PAL and C4H are considered as the essential control points of both the phenylpropanoid biosynthesis pathways. F3H is involved in the flavonoid biosynthesis pathway yielding a large family of flavonoid compounds having various biological activities including disease resistance^30^. Moreover, F3H catalyzes the production of dihydroflavonols like (2R,3R)-dihydrokaempferol and (2R,3R)-dihydroquercetin^31^ which serve as intermediates for the biosynthesis of different flavonoids^32^. Relationship between the F3H expression level and disease resistance in different host-pathogen interaction has already been reported earlier^33^.

HPLC analysis of phenolics revealed higher accumulation of flavonoids like GA, EC, ECG, EGCG and caffeine in the chitosan and CNP-treated sets. These compounds help plants to adapt to different environmental conditions and also provide resistance against pathogens by acting as feeding deterrents^34^. Treatment of litchi fruits with chitosan have been found to increase their flavonoids accumulation which apparently delayed browning and post-harvest decay effectively^35^. Flavonoids also have metal chelation properties, which limit the growth of different invasive pathogens by causing severe depletion of essential minerals^36^. The presence of EC in plant tissue may also provide a similar resistance against fungal attack^37^. Considerable increase in EC content in the chitosan and CNP treated sets might be the result of enhanced ANR expression, as ANR uses anthocyanidins as substrate to synthesize EC in the presence of reduced forms of nicotinamide adenine dinucleotide (NADH) or nicotinamide adenine dinucleotide phosphate (NADPH)^38^. These ECs are eventually converted to proanthocyanidins, which are widely distributed as plant defense compounds^39^,^40^ having profound toxicity toward pathogens^36^. Different galloylated flavonoids, which were induced significantly in the chitosan and CNP treated sets have already been reported for their antimicrobial activity^41^,^42^. Thus, the high level of flavonoid accumulation might be an indication of enhanced resistance in plants.

Exposure of plants to different biotic or abiotic stresses lead to deregulation or disruption of electric transport chain and consequently give rise to the generation of reactive oxygen species (ROS), which are considered as strong oxidizing and potentially harmful agents for the cells. With evolution, plants have evolved with different scavenging strategy in its cells including a robust antioxidant system^43^. In both the chitosan and CNP treated leaves, both enzyme activity and mRNA expression levels of SOD and CAT, two important antioxidant enzymes involved in the scavenging of ROS, were found to be significantly higher than their untreated control counterparts. This finding can be correlated with Ortega-Ortíz *et al.*^44^, who showed that the application of chitosan and salicylic acid can induce CAT activity in tomato fruits. SOD eliminates O_2_^−^ from the cells and converts them to H_2_O_2_. This H_2_O_2_ is then removed from the cells by CAT activity^45^. Hence, the increased expression of SOD and CAT due to treatment of chitosan or CNP might provide the required protection to the plants from the oxidative stress associated with pathogenic invasion.

So far several reports have highlighted the signaling role of NO, which is associated with a number of physiological processes^46^ including induction of defense mechanism in plants^12^,^15^,^47^,^48^. Involvement of NO in chitosan mediated plant defense induction has already been reported^14^,^15^,^49^. In this study, chitosan and CNP treated plants showed higher production of NO than the untreated control. NO a potent signaling molecule in plant, plays an important role in number of downstream signaling pathways^50^. To correlate the involvement of NO with CNP induced resistance, leaves of *C. sinensis* was treated with the potent NOS inhibitor, L-NAME and NO specific scavenger C-PTIO alone or in combinations. Expression of the defense related enzymes (i.e., PO, PPO, β-1,3-glucanase and PAL) including the antioxidant enzymes, SOD and CAT and the corresponding expression level of the genes (PPO, β-1,3-glucanase, PAL and TLP) and that of the phenylpropanoid biosynthesis pathway (i.e., C4H, F3H and ANR) as well as total phenol contents were slightly higher in CNP+C-PTIO and CNP+L-NAME treated sets compared to the sets treated with CNP+C-PTIO+L-NAME, C-PTIO, L-NAME and C-PTIO+L-NAME. To further confirm these findings, epidermal peals of the leaves from different treated sets were stained using the NO-specific fluoroprobe, DAF-2DA and visualized under the fluorescent microscope. Results revealed no or very little fluorescence in the NO inhibitor treated sets, whereas chitosan and CNP treated sets showed higher amount of fluorescence than the untreated control. To further prove that chitosan and CNP mediated boosting of plant defense is indeed NO-mediated, a versatile NO-donor, sodium nitroprusside (SNP) was used to create a NO surplus biological environment which witnessed a significant up-regulation of defense response in the treated leaves. Induction of defense response in rice^51^, tobacco^26^, *Capsicum annuum*^47^ by SNP application have already been reported earlier. Application of SNP have also been shown to induce the expression of SOD^52^ and CAT^53^. Hasanuzzaman and Fujita^53^ have previously reported that SNP application can also enhance the activity of different antioxidant enzymes like ascorbate peroxidase, glutathione reductase, monodehydroascorbate reductase, dehydroascorbate reductase, CAT etc. Recently, Manjunatha *et al.*^14^ and Gupta *et al.*^48^ have also demonstrated a possible relationship between the elevated level of defense responses and higher production of NO in the pearl millet in downy mildew and *Alternaria alternata* toxin-treated *Rauvolfia serpentine* callus tissue respectively. They also showed that co-treatment of chitosan and *A. alternata* toxin with L-NAME or C-PTIO reduced the NO production to basal levels in the pearl millet and *R. serpentina* callus respectively suggesting a causal relation between NO and plant defense regulation.

Although the basic role of chitosan in triggering immune responses in plants is a well-known phenomenon, our work is novel in several respects especially with regards to the exploration of chitosan nanoparticles (CNP) as a more effective therapeutic tool for disease management in plants as well as the revelation of its cellular localization and mechanism of immuno-modulatory action in plants. In fact, the role of chitosan nanoparticles (CNP) in animal models and animal cell lines are well documented^5^. However, no work has been performed thus far in the plant system with such nanoparticles. This is perhaps the first attempt to use chitosan nanoparticles for exploring its potential for disease control in plants. Our work also demonstrates for the first time the relatively higher efficacy of chitosan nanoparticles (CNP) at very low dose regimens (almost ten times less compared to that required for natural chitosan) in triggering effective immune defense responses in plants, thus unveiling the potential of chitosan nanoparticles (CNP) to act as disease control agents of choice for next generation organic cultivation. Despite extensive work on the field, no studies have been able to demonstrate the precise bio-accessibility and bio-accumulation of chitosan nanoparticles in plant cells. Here we have provided precise imaging based visual representation of the relative bio-accessibility and bio-accumulation of chitosan versus its nanoparticles (CNP) in plant cells thus uniquely demonstrating the higher level of interaction of CNP in plant cells than observed for natural chitosan – a phenomenon which helps explain why CNP is so effective in substantially lower concentration regimens compared to natural chitosan. In addition, although evidence of nitric-oxide (NO) as a versatile defense signaling molecule in plants is fast emerging, very little is known about the precise triggers that can activate such defense signaling. In this respect, elucidation of the causal role of NO in chitosan nanoparticle (CNP) mediated plant innate immune-modulation was also demonstrated for the first time through our study adding valuable information to this area of research.

In summary, it needs to be emphasized that we have mainly attempted to examine the relative efficacy of the same chitosan molecule, albeit in the form of nanoparticles in plant defense modulation, with full appreciation of the fact that a nanoparticle based delivery system may provide better response at the quantitative level of the already reported immune modulation elicited by chitosan. Indeed our demonstration of the fact that CNP has better bioaccessibility compared to chitosan and its bioaccumulation levels in plants cells is distinctly higher compared to chitosan, explains why its dose demand is substantially lower and overall efficacy is higher than natural chitosan. This implies CNP can serve as a more potent and cost-effective plant immune elicitor and replace the use of natural chitosan for future disease management in plants. Indeed, the use of CNP should be further examined under different combinations of host-pathogen interactions to establish it as a versatile anti-pathogenic or phytosanitary agent for sustainable organic cultivation in a larger scale.

## Methods

### Preparation of CNP

CNP were prepared by following the method of Qi *et al.*^10^. Chitosan was dissolved in acetic acid to a concentration of 0.5% (w/v) and the pH was adjusted to 4.6 with sodium hydroxide solution. Chitosan nanoparticles were formed spontaneously upon addition of aqueous tripolyphosphate solution (0.25%, w/v) under continuous stirring. The produced CNP were collected by centrifugation at 9000 × g. The pellet was washed multiple times to remove any sodium hydroxide and then freeze-dried before further use for analysis.

### Characterization of CNP

A Nano Size Particle Analyzer (Zen 1600 Malvern, USA) was used in the laser diffractometric analysis to measure the particle size of the CNP in the range between 0.6 nm and 6.0 mm, under the following conditions: particle refractive index 1.590, particle absorption coefficient 0.01, water refractive index 1.33 and temperature 25 °C. Transmission electron microscopic (TEM) samples of the aqueous suspension of CNP were prepared by placing a drop of the suspension on carbon-coated copper grids and allowing the water to evaporate. The micrographs were obtained by Tecnai G^2^ spirit Biotwin (FP 5018/40) TEM, operated at 80 kV accelerating voltage. For fourier transform infrared spectroscopic (FTIR) analysis, the vacuum dried CNP were mixed with potassium bromide at a ratio of 1:100 and the spectra were recorded with a FT-IR, JASCO (Japan) using a diffuse reflectance accessory under normal pH conditions (pH 7.2). The scanning data were obtained from the average of 50 scans in the range between 4000 and 400 cm^−1^.

### Treatment

*Camellia sinensis* is an extremely important crop for the economy of several countries like India, China and Sri Lanka and thus chosen as the model plant in this study. To analyze the efficacy of CNP on induction of defense response, healthy leaves of *C. sinensis* were excised and floated in different solutions containing (a) chitosan (0.01%); (b) CNP (0.001%, standardized and unpublished data). To understand the involvement of NO in the induction of defense response by CNP, the following sets were also prepared: (c) CNP (0.001%)+L-NAME (10 μM); (d) CNP (0.001%)+C-PTIO (100 μM); (e) CNP (0.001%)+L-NAME (10 μM)+C-PTIO (100 μM); (f) L-NAME (10 μM); (g) C-PTIO (100 μM); (h) L-NAME (10 μM)+C-PTIO (100 μM); (i) SNP (100 mM). Leaves floated in water alone served as control. All the sets were incubated for 24 h at ambient temperature.

### Enzyme extraction

After 24 h incubations, the leaves were homogenized with liquid nitrogen. The enzymes were extracted as described in an earlier study^12^. Five hundred mg of homogenate was extracted in 2 ml of buffer containing 0.1% polyvinylpyrrolidone and 10 μM phenylmethane sulphonyl fluoride at 4 °C. The homogenate was centrifuged at 10,500 × g for 20 min at 4 °C and the supernatants were stored at −80 °C for further use.

### Enzyme assay

PO activity was measured spectrophotometrically as described Hemeda & Klein^54^. The increase in absorbance due to formation of tetra guaiacol was recorded at 470 nm. PO activity was calculated by using the extinction coefficient of 26.6 mM^−1^ cm^−1^ for H_2_O_2_ and was expressed as *μ*mol min^−1^ g^−1^ protein. PPO activity was estimated as described by Mayer *et al.*^55^ and enzyme activity was expressed as change in absorbance at 495 nm (Δ OD change) min^−1^ g^−1^ protein. PAL was assayed following the method of Dickerson *et al.*^56^ where the conversion of L-phenylalanine to transcinnamic acid was determined spectrophotometrically at 290 nm and enzyme activity was expressed as synthesis of transcinnamic acid (n mol) min^−1^ g^−1^ protein. β-1,3-glucanase activity was estimated according to the method of Pan *et al.*^57^ with slight modifications^12^. The enzyme activity was measured spectrophotometrically at 520 nm and expressed as *μ*mol glucose equivalent produced min^−1^ g^−1^ protein. SOD was assayed by following the method described by Liang *et al.*^58^ and the enzyme activity was recorded at 560 nm. One enzyme unit (U) of SOD activity was expressed as the amount of SOD enzyme required to inhibit 50% of nitroblue tetrazolium (NBT) reduction. CAT activity was measured by following the method described by Chakraborty *et al.*^59^. The decrease in absorbance was recorded for three minutes at a wavelength of 240 nm. The enzyme activity was calculated by using the extinction coefficient of 39400 mM^−1^ cm^−1^ and was expressed as mmol min^−1^ g^−1^ of protein.

### Protein estimation

The standard Bradford assay^60^ was employed, using bovine serum albumin as a standard, to test the protein concentration of each extract.

### Semi-quantitative RT-PCR analysis

Transcript analysis of different defense related enzymes, antioxidant enzymes and related genes of flavonoid biosynthesis pathway were analyzed by semi-quantitative reverse transcription-polymerase chain reaction (RT-PCR). Total RNA was extracted with RNA-PLUS (MP Biomedicals, USA) from the tea leaves. The cDNA was synthesized from the total RNA using RT-&GO Mastermix (MP Biomedicals, USA) according to the manufacturer’s protocol. TLP, PPO, β-1,3-glucanase, PAL, C4H, F3H, ANR, CAT and SOD genes were amplified individually using gene specific primers (Table 3). Actin gene primers were used as internal control for the expression studies (Table 3). Linearity between the amount of input RNA and the final RT-PCR products was verified and confirmed. After standardizing the optimal amplification at the exponential phase, PCR cycles were carried out under the following conditions: 94 °C for 4 min, then 30 cycles of 94 °C for 30 s, annealing temperature (Tm) for specific primer for 30 s, and 72 °C for 60 s with a final extension step of 7 min at 72 °C in a thermal cycler (Applied BioSystem, USA). PCR products were subjected to electrophoresis in agarose gel and visualized under UV transilluminator, and then photographed. ImageJ software from the National Institutes of Health was used for the densitometric analysis of the photographed gels.

### Estimation of total phenol and quantification of phenolic compounds by HPLC

Total phenolic content of the control and different treated leaves were estimated according to Zieslin and Ben-Zaken^61^. Phenolic content was measured spectrophotometrically at 725 nm using gallic acid as a standard. The amount of phenolics was expressed as *μ*g gallic acid g^−1^ fresh weight of leaves. Samples prepared from tea leaves for phenolic estimation were analyzed with an HPLC system (Agilent, USA) equipped with an Agilent DAAD detector and an Agilent Eclipse plus C18 column (100 mm × 4.6 mm, 3.5 μm) as described earlier^12^. Eight compounds, namely, gallic acid (GA), caffeine, epicatechin (EC), epigallocatechin (EGC), epigallocatechin gallate (EGCG), epicatechin gallate (ECG), gallocatechin gallate (GCG), and catechin gallate (CG) were detected in samples and there concentrations were calculated by comparing peak areas of the individual reference standards that were run under the same elution conditions.

### Nitric oxide Estimation

Rate of NO production by 24 h incubated leaves were estimated by hemoglobin assay^62^. Production of NO was measured spectrophotometrically at 401 nm at an hourly basis and NO levels were calculated using an extinction coefficient of 38,600 M^−1^ cm^−1^ 63. NO content in the reaction mixture was measured in terms of nmol of NO produced g^−1^ fresh weight of leaves h^−1^ and compared with the water control.

Real-time NO production was visualized using the membrane permeable NO-specific fluoroprobe, 4-5-diaminofluorescein diacetate (DAF-2DA) dye^64^. The lower epidermis of the leaves were peeled off and then incubated in DAF-2DA solution for 20 min in the dark. Fluorescence was observed with a Leica DMLS microscope at an excitation wavelength of 480 nm and emission wavelength of 500–600 nm.

### Preparation of fluorescent tagged chitosan and CNP and assessment of their bioaccumulation

FITC is commonly used to label the amino functional group of proteins to allow quantification by fluorometric methods and visualization under the confocal microscope. The synthesis of FITC-labeled chitosan was carried out following the method of Huang *et al.*^65^. 1 g of chitosan was dissolved in 100 ml of 0.10 M acetic acid and gradually 100 ml of dehydrated methanol was added to it with continuous stirring, followed by the addition of 50 ml of FITC dissolved in methanol at 2.0 mg/ml. The reaction between the isothiocyanate group of FITC and the primary amino group of the D-glucosamine residue was allowed to proceed for 3 h in the dark at ambient conditions. Yellow colored FITC-labeled chitosan (CHT-FITC) was precipitated in 0.2 M NaOH, and washed with 70% methanol until the supernatant becomes free from fluorescence. FITC-labeled chitosan nanoparticles (CNP-FITC) were prepared in the same way, under the same conditions as above using unlabeled chitosan nanoparticles as the starting material.

To test the whether the FITC labeled chitosan and CNP could penetrate the rigid plant cell wall, healthy leaves of *C. sinensis* were excised and floated in the CHT-FITC (0.01%) and CNP-FITC (0.001%) solutions respectively for 24 h. For control experiments, leaves were incubated in water along with FITC under the same condition mentioned above. After the completion of treatment lower epidermis of the leaves were peeled off from both the control and treated sets and observed under the fluorescent microscope.

### Statistical Analysis

All data presented were the mean ± SD of three separate experiments conducted in triplicate. In all the experiments, the data obtained were subjected to analysis of variance (ANOVA) using SPSS software version 20 and the significance of difference between the treatments was determined using Duncan’s Multiple Range Test (*p* < *0.05*).

## Additional Information

**How to cite this article**: Chandra, S. *et al.* Chitosan nanoparticles: A positive modulator of innate immune responses in plants. *Sci. Rep.* **5**, 15195; doi: 10.1038/srep15195 (2015).

## Figures and Tables

**Table 1:** Effect of exogenous treatment of Chitosan (0.1%), CNP (0.01%), SNP (100 μM), C-PTIO (100 μM) and L-NAME ((10 μM) alone or in different combinations on the production of defense enzymes and total phenol in detached leaves of *C. sinensis*.

	Control	Chitosan	CNP	SNP	CNP+C-PTIO	CNP+L-NAME	CNP+C-PTIO+L-NAME	C-PTIO	L-NAME	C-PTIO+L-NAME
PO [μmol min^−1^ g^−1^ protein]	9.19 ± 1.11^*d*^	28.13 ± 1.74^*b*^	37.91 ± 1.18^*a*^	38.28 ± 2.46^*a*^	11.78 ± 1.64^*c*^	11.23 ± 0.15^*cd*^	9.29 ± 1.01^*d*^	5.81 ± 0.76^*e*^	5.99 ± 0.93^*e*^	5.96 ± 0.52^*e*^
PPO [(Δ OD change) min^−1^ g^−1^ protein]	4.07 ± 0.34^*c*^	9.78 ± 0.89^*b*^	14.33 ± 1.66^*a*^	15.15 ± 0.76^*a*^	4.67 ± 0.48^*c*^	4.64 ± 0.32^*c*^	4.33 ± 0.44^*c*^	3.57 ± 0.51^*c*^	3.56 ± 0.85^*c*^	3.58 ± 0.14^*c*^
β-1,3-glucanase [μmol glucose produced min^−1^ g^−1^ protein]	97.41 ± 4.52^*c*^	125.91 ± 3.29^*b*^	131.03 ± 3.9^*ab*^	135.98 ± 4.98^*a*^	88.33 ± 5.16^*d*^	88.27 ± 4.5^*d*^	85.24 ± 4.61^*de*^	78.55 ± 8.93^*ef*^	75.78 ± 3.03^*f*^	70.34 ± 5.61^*f*^
PAL [n mol of transcinnamic acid min^−1^ g^−1^ protein]	8.26 ± 0.33^*d*^	15.2 ± 0.22^*c*^	17.36 ± 0.57^*b*^	18.94 ± 1.06^*a*^	8.43 ± 0.42^*d*^	8.39 ± 0.33^*d*^	8.24 ± 0.34^*d*^	6.51 ± 0.47^*e*^	6.02 ± 0.59^*e*^	5.9 ± 0.37^*e*^
SOD [Units min^−1^ g^−1^ protein]	15.13 ± 2.37^*c*^	22.78 ± 1.47^*b*^	25.53 ± 0.80^*a*^	26.46 ± 1.37^*a*^	16.70 ± 1.84^*c*^	16.58 ± 2.27^*c*^	15.10 ± 1.79^*c*^	12.01 ± 0.74^*d*^	11.38 ± 1.16^*d*^	11.02 ± 0.92^*d*^
CAT [m mol min^−1^ g^−1^ protein]	343.51 ± 35.83^*c*^	573.47 ± 43.09^*b*^	667.73 ± 23.89^*a*^	635.71 ± 59.05^*a*^	364.26 ± 29.54^*c*^	361.42 ± 23.41^*c*^	359.36 ± 44.06^*c*^	249.26 ± 26.81^*d*^	247.14 ± 41.06^*d*^	230.54 ± 18.42^*d*^
Phenol [μg gallic acid g^−1^ tissue]	901.67 ± 2.6^*def*^	1082.5 ± 12.05^*c*^	1121.25 ± 13.75^*b*^	1181.25 ± 8.75^*a*^	918.75 ± 12.31^*d*^	916.67 ± 12.52^*d*^	906.25 ± 7.5^*de*^	890.42 ± 8.86^*efg*^	887.08 ± 8.78^*fg*^	872.92 ± 5.91^*g*^

Results are mean ± SD of three separate experiments done in triplicate. Different letters within the row indicate significant difference (*p* < *0.05*) from the control set using Duncan’s multiple range test. Same letter within the row denotes no significant difference between the groups. *CNP: chitosan nanoparticles; SNP: sodium nitroprusside; C-PTIO: 2-(4-Carboxyphenyl)-4, 4, 5, 5-tetramethylimidazoline-1-oxyl-3-oxide; L-NAME: NG-nitro-L-arginine methyl ester; PO: peroxidase; PPO: polyphenol oxidase; PAL: phenylalanine ammonia lyase; SOD: superoxide dismutase; CAT: catalase.*

**Table 2:** Quantitative changes in the phenolic acid content in excised leaves of *C. sinensis* due to the application of Chitosan (0.1%), CNP (0.01%), SNP (100 μM), C-PTIO (100 μM) and L-NAME (10 μM) alone or in different combinations.

Identifiedcompounds	Quantity (μg/g of fresh weight)										
	Control	CHT	CNP	SNP	CNP+C-PTIO	CNP+L-NAME	CNP+C-PTIO+L-NAME	C-PTIO	L-NAME	C-PTIO+L-NAME	
GA	4.44 ± 0.39^*c*^	5.52 ± 0.15^*ab*^	5.97 ± 0.77^*a*^	5.85 ± 0.37^*a*^	4.95 ± 0.37^*c*^	4.95 ± 0.17^*bc*^	4.91 ± 0.14^*bc*^	4.47 ± 0.28^*c*^	4.42 ± 0.28^*c*^	4.30 ± 0.23^*c*^	
EGC	13.41 ± 0.35^*a*^	13.66 ± 0.47^*a*^	13.25 ± 0.27^*a*^	13.54 ± 0.21^*a*^	13.46 ± 0.26^*a*^	13.24 ± 0.24^*a*^	13.28 ± 0.33^*a*^	13.34 ± 0.76^*a*^	13.45 ± 0.47^*a*^	13.7 ± 0.27^*a*^	
CAF	55.89 ± 1.35^*d*^	74.39 ± 2.93^*b*^	77.94 ± 2.24^*a*^	80.97 ± 1.34^*a*^	63.58 ± 2.08^*c*^	62.36 ± 2.07^*c*^	60.34 ± 2.74^*c*^	50.74 ± 1.91^*e*^	50.21 ± 1.18^*e*^	47.88 ± 2.11^*e*^	
EC	7.33 ± 0.44^*b*^	8.34 ± 0.31^*a*^	8.45 ± 0.44^*a*^	8.31 ± 0.37^*a*^	7.21 ± 0.11^*b*^	7.14 ± 0.14^*bc*^	7.11 ± 0.21^*bc*^	6.78 ± 0.24^*bc*^	6.72 ± 0.3^*bc*^	6.49 ± 0.59^*c*^	
EGCG	154.55 ± 4.67^*d*^	174.46 ± 5.42^*b*^	186.77 ± 5.47^*a*^	184.78 ± 4.29^*a*^	165.87 ± 3.65^*c*^	163.18 ± 3.60^*c*^	153.91 ± 3.65^*de*^	146.09 ± 3.72^*f*^	147.07 ± 3.43^*ef*^	144.01 ± 3.13^*f*^	
GCG	5.20 ± 0.86^*a*^	5.69 ± 0.63^*a*^	5.1 ± 0.15^*a*^	5.65 ± 0.7^*a*^	5.27 ± 0.36^*a*^	5.46 ± 0.49^*a*^	5.26 ± 0.36^*a*^	5.17 ± 0.97^*a*^	5.57 ± 0.51^*a*^	5.02 ± 0.34^*a*^	
ECG	56.69 ± 3.02^*c*^	72.02 ± 3.52^*b*^	85.58 ± 4.35^*a*^	82.83 ± 4.35^*a*^	55.57 ± 1.27^*c*^	54.55 ± 2.68^*cd*^	56.00 ± 2.22^*c*^	49.95 ± 1.87^*de*^	47.03 ± 2.99^*e*^	46.46 ± 1.06^*e*^	
CG	1.7 ± 0.38^*a*^	1.56 ± 0.43^*a*^	1.77 ± 0.32^*a*^	1.7 ± 0.33^*a*^	1.94 ± 0.12^*a*^	1.73 ± 0.35^*a*^	1.54 ± 0.48^*a*^	1.67 ± 0.49^*a*^	1.76 ± 0.36^*a*^	1.83 ± 0.35^*a*^	

Results are mean ± SD of three separate experiments, done in triplicate. Different letters within the row indicate significant difference (*p* < *0.05*) from the control set using Duncan’s multiple range test. Same letter within the row denotes no significant difference between the groups. *CNP: chitosan nanoparticles; SNP: sodium nitroprusside; C-PTIO: 2-(4-Carboxyphenyl)-4, 4, 5, 5-tetramethylimidazoline-1-oxyl-3-oxide; L-NAME: NG-nitro-L-arginine methyl ester; GA*: *gallic acid; EGC: epigallocatechin; EC: epicatechin; EGCG: epigallocatechin gallate; GCG: gallocatechin gallate; ECG: epicatechin gallate; CG: catechin gallate.*

**Table 3:** Primer sequences used in RT-PCR analyses.

Gene of interest	Sequences of primers	Tm (°C)	References
TLP	FP: 5′ GACCAACTGCAACTTCGATGCCAA 3′	60	12
	RP: 5′ AAGCATCTGGGCACCTATCCTTGA 3′		
PPO	FP: 5′ TACCCGCCATGTATGCGGACATAA 3′	68	(ACCESSION KJ808721)
	RP: 5′ ATGGCAGCAGAATCCAGTGTCTCT 3′		
β-1,3-glucanase	FP: 5′ TGCTTCTTGCACTTGCCAATACCG 3′	68	(ACCESSION KJ808722)
	RP: 5′ ACTGACTAGGACGTGTGCTGCATT 3′		
PAL	FP: 5′ GCCGAGCAACATAACCAAG 3′	61	66
	RP: 5′ ACAGATAGGAAGAGGAGCAC 3′		
C4H	FP: 5′ CGCGTGGTGGCTTGCAAACAACCCC 3′	60	66
	RP: 5′ TGGCCTGTCCCGGAGGAGGCAAGAG 3′		
F3H	FP: 5′ GAGATGACTCGACTCGCTCGTGAGTTT 3′	60	67
	RP: 5′ TAGCCTCAGACAACACCTCCAGCAACT 3′		
ANR	FP: 5′ CACCTTCTAGCACTAAAGGGTTCAGG 3′	55	68
	RP: 5′ GTGTCAGTCCAGTGACTCTCATCCAT 3′		
CAT	FP: 5′ AAGACTACAGGCACATGGAAGGCT 3′	60	12
	RP: 5′ ACCAGGTACAATGATAGCAGGGCA 3′		
SOD	FP: 5′ CAAGAAGGAGATGGTCCAACTACAGTTACC 3′	61	69
	RP: 5′ AACAACAACAGCCCTTCCAATGACG 3′		
Actin	FP: 5′ TGAGAGATTCCGTTGCCCTGAAGT 3′	61	12
	RP: 5′ AACCTGTTGGAAGGTGCTTAGGGA 3′		

TLP: thaumatin like protein; PPO: polyphenol oxidase; PAL: phenylalanine ammonia lyase; C4H: cinnamate 4-hydroxylase; F3H: flavonoid 3-hydroxylase; ANR: anthocyanidin reductase; CAT: catalase; SOD: superoxide dismutase.

## References

[b1] Saharan V. *et al.* Synthesis of chitosan based nanoparticles and their *in vitro* evaluation against phytopathogenic fungi. Int. J. Biol. Macromol. 62, 677–683 (2013). 24141067 10.1016/j.ijbiomac.2013.10.012

[b2] Saharan V. *et al.* Synthesis and *in vitro* antifungal efficacy of Cu–chitosan nanoparticles against pathogenic fungi of tomato. Int. J. Biol. Macromol. 75, 346–353 (2015). 25617841 10.1016/j.ijbiomac.2015.01.027

[b3] El Hadrami A., Adam L. R., El Hadrami I. & Daayf F. Chitosan in plant protection. Mar. Drugs 8, 968–987 (2010). 20479963 10.3390/md8040968 PMC2866471

[b4] Shukla S. K., Mishra A. K., Arotiba O. A. & Mamba B. B. Chitosan-based nanomaterials: A state-of-the-art review. Int. J. Biol. Macromol. 59, 46–58 (2013). 23608103 10.1016/j.ijbiomac.2013.04.043

[b5] Nagpal K., Singh S. K. & Mishra D. N. Chitosan nanoparticles: a promising system in novel drug delivery. Chem. Pharm. Bull. (Tokyo). 58, 1423–1430 (2010). 21048331 10.1248/cpb.58.1423

[b6] Rahaman Mollick M. M. *et al.* Anticancer (*in vitro*) and antimicrobial effect of gold nanoparticles synthesized using Abelmoschus esculentus (L.) pulp extract via a green route. RSC Adv. 4, 37838 (2014).

[b7] Banerjee T., Mitra S., Kumar Singh A., Kumar Sharma R. & Maitra A. Preparation, characterization and biodistribution of ultrafine chitosan nanoparticles. Int. J. Pharm. 243, 93–105 (2002). 12176298 10.1016/s0378-5173(02)00267-3

[b8] Sarkar J., Ray S., Chattopadhyay D., Laskar A. & Acharya K. Mycogenesis of gold nanoparticles using a phytopathogen *Alternaria alternata*. Bioprocess Biosyst. Eng. 35, 637–643 (2012). 22009439 10.1007/s00449-011-0646-4

[b9] Jia-hui Y., Yu-min D. & Hua Z. Blend films of chitosan-gelatin. Wuhan Univ. J. Nat. Sci. 4, 476 (1999).

[b10] Qi L., Xu Z., Jiang X., Hu C. & Zou X. Preparation and antibacterial activity of chitosan nanoparticles. Carbohydr. Res. 339, 2693–2700 (2004). 15519328 10.1016/j.carres.2004.09.007

[b11] Nimal Punyasiri P. A. *et al.* *Exobasidium vexans* infection of *Camellia sinensis* increased 2,3-cis isomerisation and gallate esterification of proanthocyanidins. Phytochemistry 65, 2987–2994 (2004). 15504433 10.1016/j.phytochem.2004.09.004

[b12] Chandra S. *et al.* Abiotic elicitor-mediated improvement of innate immunity in *Camellia sinensis*. J. Plant Growth Regul. 33, 849–859 (2014).

[b13] Hammerbacher A. *et al.* Flavan-3-ols in Norway Spruce: biosynthesis, accumulation, and function in response to attack by the bark beetle-associated fungus *Ceratocystis polonica*. Plant Physiol. 164, 2107–22 (2014). 24550241 10.1104/pp.113.232389 PMC3982766

[b14] Manjunatha G. *et al.* Nitric oxide is involved in chitosan-induced systemic resistance in pearl millet against downy mildew disease. Pest Manag. Sci. 65, 737–743 (2009). 19222022 10.1002/ps.1710

[b15] Acharya K., Chakraborty N., Dutta A. K., Sarkar S. & Acharya R. Signaling role of nitric oxide in the induction of plant defense by exogenous application of abiotic inducers. Arch. Phytopathol. Plant Prot. 44, 1501–1511 (2011).

[b16] Sánchez-Estrada A., Tiznado-Hernández M. E., Ojeda-Contreras A. J., Valenzuela-Quintanar A. I. & Troncoso-Rojas R. Induction of enzymes and phenolic compounds related to the natural defence response of netted melon fruit by a bio-elicitor. J. Phytopathol. 157, 24–32 (2009).

[b17] McCann H. C., Nahal H., Thakur S. & Guttman D. S. Identification of innate immunity elicitors using molecular signatures of natural selection. Proc. Natl. Acad. Sci. 109, 4215–4220 (2012). 22323605 10.1073/pnas.1113893109 PMC3306723

[b18] Hadwiger L. A. Plant science review: Multiple effects of chitosan on plant systems: Solid science or hype. Plant Sci. 208, 42–49 (2013). 23683928 10.1016/j.plantsci.2013.03.007

[b19] Lamb C. & Dixon R. A. The oxidative burst in plant disease resistance. Annu. Mol.Biol. 48, 251–275 (1997). 10.1146/annurev.arplant.48.1.251 15012264

[b20] Bruce R. J. & West C. A. Elicitation of lignin biosynthesis and isoperoxidase activity by pectic fragments in suspension cultures of castor bean. Plant Physiol. 91, 889–897 (1989). 16667153 10.1104/pp.91.3.889 PMC1062092

[b21] Balasubramanian V., Vashisht D., Cletus J. & Sakthivel N. Plant β-1,3-glucanases: Their biological functions and transgenic expression against phytopathogenic fungi. Biotechnol. Lett. 34, 1983–1990 (2012). 22850791 10.1007/s10529-012-1012-6

[b22] Iriti M. & Varoni E. M. Chitosan-induced antiviral activity and innate immunity in plants. Environ. Sci. Pollut. Res. 22, 2935–2944 (2014). 10.1007/s11356-014-3571-7 25226839

[b23] El Hassni M., El Hadrami A., Daayf F., Barka E. A. & El Hadrami I. Chitosan, antifungal product against *Fusarium oxysporum* f. sp. albedinis and elicitor of defence reactions in date palm roots. Phytopathol. Mediterr. 43, 195–204 (2004).

[b24] Romanazzi G., Nigro F., Ippolito A., Di Venere D. & Salerno M. Effects of pre and postharvest chitosan treatments to control storage grey mold of table grapes. J. Food Sci. 67, 1862–1867 (2002).

[b25] Fajardo J. E., McCollum T. G., McDonald R. E. & Mayer R. T. Differential induction of proteins in orange flavedo by biologically based elicitors and challenged by *Penicillium digitatum*. Sacc.1. Biol. Control 13, 143–151 (1998).

[b26] Zhao X. M., She X. P., Yu W., Liang X. M. & Du Y. G. Effects of oligochitosans on tobacco cells and role of endogenous nitric oxide burst in the resistance of tobacco to Tobacco mosaic virus. J. Plant Pathol. 89, 55–65 (2007).

[b27] Pellegrini L., Rohfritsch O., Fritig B. & Legrand M. Phenylalanine ammonia-lyase in tobacco. Plant Physiol. 106, 877–886 (1994). 7824656 10.1104/pp.106.3.877 PMC159610

[b28] Nicholson R. L. & Hammerschmidt R. Phenolic compounds and their role in disease resistance. Annu. Rev. Phytopathol. 30, 369–389 (1992).

[b29] Dong J., Wan G. & Liang Z. Accumulation of salicylic acid-induced phenolic compounds and raised activities of secondary metabolic and antioxidative enzymes in *Salvia miltiorrhiza* cell culture. J. Biotechnol. 148, 99–104 (2010). 20576504 10.1016/j.jbiotec.2010.05.009

[b30] Khlestkina E. K. *et al.* Comparative molecular marker-based genetic mapping of flavanone 3-hydroxylase genes in wheat, rye and barley. Euphytica 179, 333–341 (2011).

[b31] Britsch L. & Grisebach H. Purification and characterization of (2S)-flavanone 3-hydroxylase from *Petunia hybrida*. Eur. J. Biochem. 156, 569–577 (1986). 3699024 10.1111/j.1432-1033.1986.tb09616.x

[b32] Holton T. & Cornish E. Genetics and biochemistry of anthocyanin biosynthesis. Plant Cell 7, 1071–1083 (1995). 12242398 10.1105/tpc.7.7.1071 PMC160913

[b33] Giovanini M. P. *et al.* Gene-for-Gene defense of wheat against the hessian fly lacks a classical oxidative burst. Mol. Plant-Microbe Interact. 19, 1023–1033 (2006). 16941906 10.1094/MPMI-19-1023

[b34] Gould K. & Lister C. in Flavonoids 397–441 (CRC Press, 2006), 10.1201/9781420039443.ch8.

[b35] Zhang D. & Quantick P. C. Effects of chitosan coating on enzymatic browning and decay during postharvest storage of litchi (*Litchi chinensis* Sonn.) fruit. Postharvest Biol. Technol. 12, 195–202 (1997).

[b36] Scalbert A. Antimicrobial properties of tannins. Phytochemistry 30, 3875–3883 (1991).

[b37] Aron P. M. & Kennedy J. A. Flavan-3-ols: Nature, occurrence and biological activity. Mol. Nutr. Food Res. 52, 79–104 (2008). 18081206 10.1002/mnfr.200700137

[b38] Xie D.-Y., Sharma S. B., Paiva N. L., Ferreira D. & Dixon R. A. Role of anthocyanidin reductase, encoded by BANYULS in plant flavonoid biosynthesis. Sci. 299, 396–399 (2003). 10.1126/science.1078540 12532018

[b39] He F., Pan Q. H., Shi Y. & Duan C. Q. Biosynthesis and genetic regulation of proanthocyanidins in plants. Molecules 13, 2674–2703 (2008). 18971863 10.3390/molecules13102674 PMC6245171

[b40] Mierziak J., Kostyn K. & Kulma A. Flavonoids as important molecules of plant interactions with the environment. Molecules 19, 16240–16265 (2014). 25310150 10.3390/molecules191016240 PMC6270724

[b41] Daglia M. Polyphenols as antimicrobial agents. Curr. Opin. Biotechnol. 23, 174–181 (2012). 21925860 10.1016/j.copbio.2011.08.007

[b42] Sourabh A., Kanwar S. S., Sud R. G., Ghabru A. & Sharma O. P. Influence of phenolic compounds of Kangra tea [*Camellia sinensis* (L) O Kuntze] on bacterial pathogens and indigenous bacterial probiotics of Western Himalayas. Brazilian J. Microbiol. 715, 709–715 (2013). 10.1590/s1517-83822013000300007 PMC3910178 24516437

[b43] Groß F., Durner J. & Gaupels F. Nitric oxide, antioxidants and prooxidants in plant defence responses. Front. Plant Sci. 4, 419 (2013). 24198820 10.3389/fpls.2013.00419 PMC3812536

[b44] Ortega-Ortíz H., Benavides-Mendoza A., Mendoza-Villarreal R., Ramírez-Rodríguez H. & de Alba-Romenus K. Enzymatic activity in tomato fruits as a response to chemical elicitors. J. Mex. Chem. Soc. 51, 141–144 (2007).

[b45] Yildiztugay E., Ozfidan-Konakci C. & Kucukoduk M. Modulation of osmotic adjustment and antioxidant status in salt-stressed leaves of *Thermopsis turcica*. Acta Physiol. Plant. 36, 125–138 (2014).

[b46] Baudouin E. The language of nitric oxide signalling. Plant Biol. 13, 233–242 (2011). 21309969 10.1111/j.1438-8677.2010.00403.x

[b47] Acharya K., Chandra S., Chakraborty N. & Acharya R. Nitric oxide functions as a signal in induced systemic resistance. Arch. Phytopathol. Plant Prot. 44, 1335–1342 (2011).

[b48] Gupta N. S., Banerjee M., Basu S. K. & Acharya K. Involvement of nitric oxide signal in *Alternaria alternata* toxin induced defense response in *Rauvolfia serpentina* Benth. ex Kurz calli. Plant Omi. J. 6, 157–164 (2013).

[b49] Terrile M. C., Mansilla A. Y., Albertengo L., Rodríguez M. S. & Casalongué C. A. Nitric-oxide-mediated cell death is triggered by chitosan in *Fusarium eumartii* spores. Pest Manag. Sci. 71, 668–674 (2015). 24764137 10.1002/ps.3814

[b50] Perchepied L. *et al.* Nitric oxide participates in the complex interplay of defense-related signaling pathways controlling disease resistance to *Sclerotinia sclerotiorum* in *Arabidopsis thaliana*. Mol. Plant-Microbe Interact. 23, 846–860 (2010). 20521948 10.1094/MPMI-23-7-0846

[b51] Hu X., Neill S. J., Cai W. & Tang Z. NO-mediated hypersensitive responses of rice suspension cultures induced by incompatible elicitor. Chinese Sci. Bull. 48, 358–363 (2003).

[b52] Singh H. P., Batish D. R., Kaur G., Arora K. & Kohli R. K. Nitric oxide (as sodium nitroprusside) supplementation ameliorates Cd toxicity in hydroponically grown wheat roots. Environ. Exp. Bot. 63, 158–167 (2008).

[b53] Hasanuzzaman M. & Fujita M. Exogenous sodium nitroprusside alleviates arsenic-induced oxidative stress in wheat (*Triticum aestivum* L.) seedlings by enhancing antioxidant defense and glyoxalase system. Ecotoxicology 22, 584–596 (2013). 23430410 10.1007/s10646-013-1050-4

[b54] Hemeda H. M. & Klein B. P. Effects of naturally occurring antioxidants on peroxidase activity of vegetable extracts. J. Food Sci. 55, 184–185 (1990).

[b55] Mayer A. M., Harel E. & Ben-Shaul R. Assay of catechol oxidase—a critical comparison of methods. Phytochemistry 5, 783–789 (1966).

[b56] Dickerson D. P., Pascholati S. F., Hagerman A. E., Butler L. G. & Nicholson R. L. Phenylalanine ammonia-lyase and hydroxycinnamate: CoA ligase in maize mesocotyls inoculated with *Helminthosporium maydis* or *Helminthosporium carbonum*. Physiol. Plant Pathol. 25, 111–123 (1984).

[b57] Pan S. Q., Ye X. S. & Kuć J. Association of β-1,3-glucanase activity and isoform pattern with systemic resistance to blue mould in tobacco induced by stem injection with *Peronospora tabacina* or leaf inoculation with tobacco mosaic virus. Physiol. Mol. Plant Pathol. 39, 25–39 (1991).

[b58] Liang J. G., Tao R. X., Hao Z. N., Wang L. P. & Zhang X. Induction of resistance in cucumber against seedling damping-off by plant growth-promoting rhizobacteria (PGPR) *Bacillus megaterium* strain L8. African J. Biotechnol. 10, 6920–6927 (2011).

[b59] Chakraborty N., Chandra S. & Acharya K. Sublethal heavy metal stress stimulates innate immunity in tomato. Sci. World J. Article ID (2014). 10.1155/2015/208649 PMC4333193 25729768

[b60] Bradford M. M. A rapid and sensitive method for the quantitation of microgram quantities of protein utilizing the principle of protein-dye binding. Anal. Biochem. 72, 248–254 (1976). 942051 10.1016/0003-2697(76)90527-3

[b61] Zieslin N. & Ben-zaken R. Peroxidase activity and presence of phenolic substances in peduncles of rose flowers. Plant Physiol. Biochem. 31, 333–339 (1993).

[b62] Delledonne M., Xia Y., Dixon R. A. & Lamb C. Nitric oxide functions as a signal in plant disease resistance. Nature 394, 585–588 (1998). 9707120 10.1038/29087

[b63] Salter M. & Knowles R. in Nitric Oxide Protocols SE—6 (ed. Titheradge M.) 100, 61–65 (Humana Press, 1998).

[b64] Bartha B., Kolbert Z. & Erdei L. Nitric oxide production induced by heavy metals in *Brassica juncea* L. Czern and *Pisum* L. Acta Biol. Szeged. 49, 9–12 (2005).

[b65] Huang M., Khor E. & Lim L.-Y. Uptake and cytotoxicity of chitosan molecules and nanoparticles: effects of molecular weight and degree of deacetylation. Pharm. Res. 21, 344–353 (2004). 15032318 10.1023/b:pham.0000016249.52831.a5

[b66] Singh K., Kumar S., Rani A., Gulati A. & Ahuja P. S. Phenylalanine ammonia-lyase (PAL) and cinnamate 4-hydroxylase (C4H) and catechins (flavan-3-ols) accumulation in tea. Funct. Integr. Genomics 9, 125–134 (2009). 18679731 10.1007/s10142-008-0092-9

[b67] Singh K. *et al.* An early gene of the flavonoid pathway, flavanone 3-hydroxylase, exhibits a positive relationship with the concentration of catechins in tea (*Camellia sinensis*). Tree Physiol. 28, 1349–1356 (2008). 18595847 10.1093/treephys/28.9.1349

[b68] Singh K. *et al.* Differential display mediated cloning of anthocyanidin reductase gene from tea (*Camellia sinensis*) and its relationship with the concentration of epicatechins. Tree Physiol. 29, 837–846 (2009). 19380395 10.1093/treephys/tpp022

[b69] Das A., Das S. & Mondal T. K. Identification of differentially expressed gene profiles in young roots of tea [*Camellia sinensis* (L.) O. Kuntze] subjected to drought stress using suppression subtractive hybridization. Plant Mol. Biol. Report. 30, 1088–1101 (2012).

